# Characteristics, survival, and risk factors of Chinese young lung cancer patients: the experience from two institutions

**DOI:** 10.18632/oncotarget.19183

**Published:** 2017-07-12

**Authors:** Jianjie Li, Fan Yang, Xiao Li, Min Zhang, Ruozi Fu, Xiaodan Yin, Jun Wang

**Affiliations:** ^1^ Department of Pulmonary Oncology, 307 Hospital of the Academy of Military Medical Sciences, Beijing, China; ^2^ Department of Thoracic Surgery, Peking University People’s Hospital, Beijing, China

**Keywords:** lung cancer, survival, incidence, risk factors, China

## Abstract

Limited data is available regarding lung cancer in Chinese young adults. This study was aimed to determine the characteristics, survival, and prognostic factors of young lung cancer patients in China. We reviewed demographic and clinical data, and survival information of 420 young patients (20–45 years old) diagnosed with lung cancer in two Chinese hospitals between 2000 and 2013. The results showed that lung cancer occurred more frequently (70%) and affected more males than females (43.8% *vs* 26.7%) in patients older than 36; whereas, more females were affected under age 35 (16.7% *vs* 12.8%). Most patients had adenocarcinoma (67.6%) and stage IV disease (72.4%) at presentation. The median survival of all young patients with lung cancer was 44 months (95% CI: 39–49), of which patients with stage I–IIIA disease had a longer survival than those with stage IIIB/IV disease (101 *vs* 22 months, *p* < 0.001). No significant difference in survival was found in patients having different histological subtypes or genders. Multivariate analysis revealed that high exposure risk occupations, smoking, family history of lung cancer were risk factors of young lung cancer patients. This study provides an overview of the clinical characteristics, patterns and prognostic factors of young patients with lung cancer in China.

## INTRODUCTION

Lung cancer has become a serious health burden worldwide, accounting for more than one-quarter (27%) of all cancer-related deaths, with an estimated 2.2 million new cases and 1.6 million deaths per year [1]. During the past decades, the incidence and mortality of lung cancer in China are increasing sharply, with an estimated 733300 newly diagnosed cases and 610200 deaths in each year [2–4].

The risk of developing lung cancer is associated with many factors such as age. In 2005-2009, about 71% of non-small cell lung cancer (NSCLC) patients were aged over 70 years, and more than one third of these patients were 80 years or older [5]. Lung cancer in young patients (≤ 45 years old) is less common and has different clinical characteristics as compared with older patients. In recent years, there was a rapid increase in incidence of lung cancer among young population. A retrospective study reviewed by British scientists using the National Lung Cancer Audit database between 2004 and 2011 has reported that young lung cancer patients were accounted for about 0.5% of the overall lung cancer population, whose performance score and survival were better than older lung cancer patients [6]. A study conducted in the United States has evaluated the surveillance, epidemiology and end results data of lung cancer between 1998 and 2003, and reported that young lung cancer patients were accounted for 1.17% of the total population and had a greater representation of African Americans, Asians, women, and adenocarcinoma histology compared with the older cohort [7]. There is a trend of increasing incidence of younger patients in China. A Chinese study reported the incidence of young lung cancer population was 5.275% of overall lung cancer population [8]. Risk factors of lung cancer in the general population include smoking status, air pollution, exposure to carcinogens (e.g. asbestos), and ionizing radiation [9–12], however, data regarding on young lung cancer patient subgroup are limited.

The treatment, prognosis, and outcomes of young lung cancer patients in China have not been fully explored; there is an urgent need to identify the key characteristics and more information of lung cancer in this specific population. Here we present a retrospective study performed in China to investigate the trend, characteristics, survival, and potential risk factors of lung cancer patients younger than 45 years.

## RESULTS

**Figure 1:**
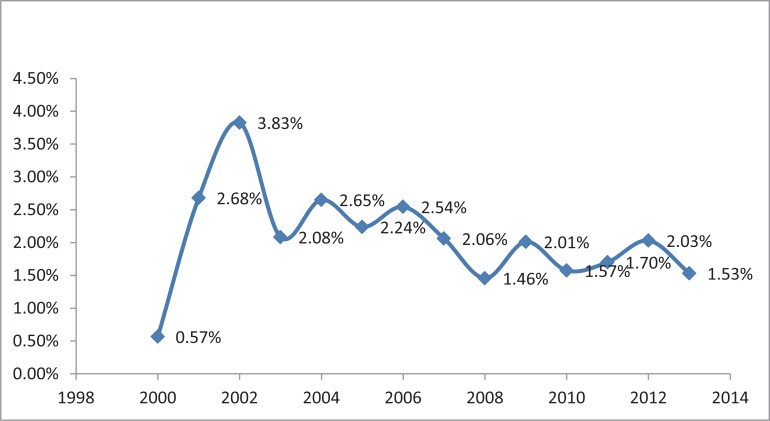
Percentage of lung cancer in overall population

A total of 420 (1.84%) young lung cancer patients were enrolled in this study out of 22820 lung cancer patients of all age. The percentage of young patients increased from 0.57% to 3.83% between 2000 and 2002, and slight decreased from 2002 to 2013 (Figure 1).

**Figure 2:**
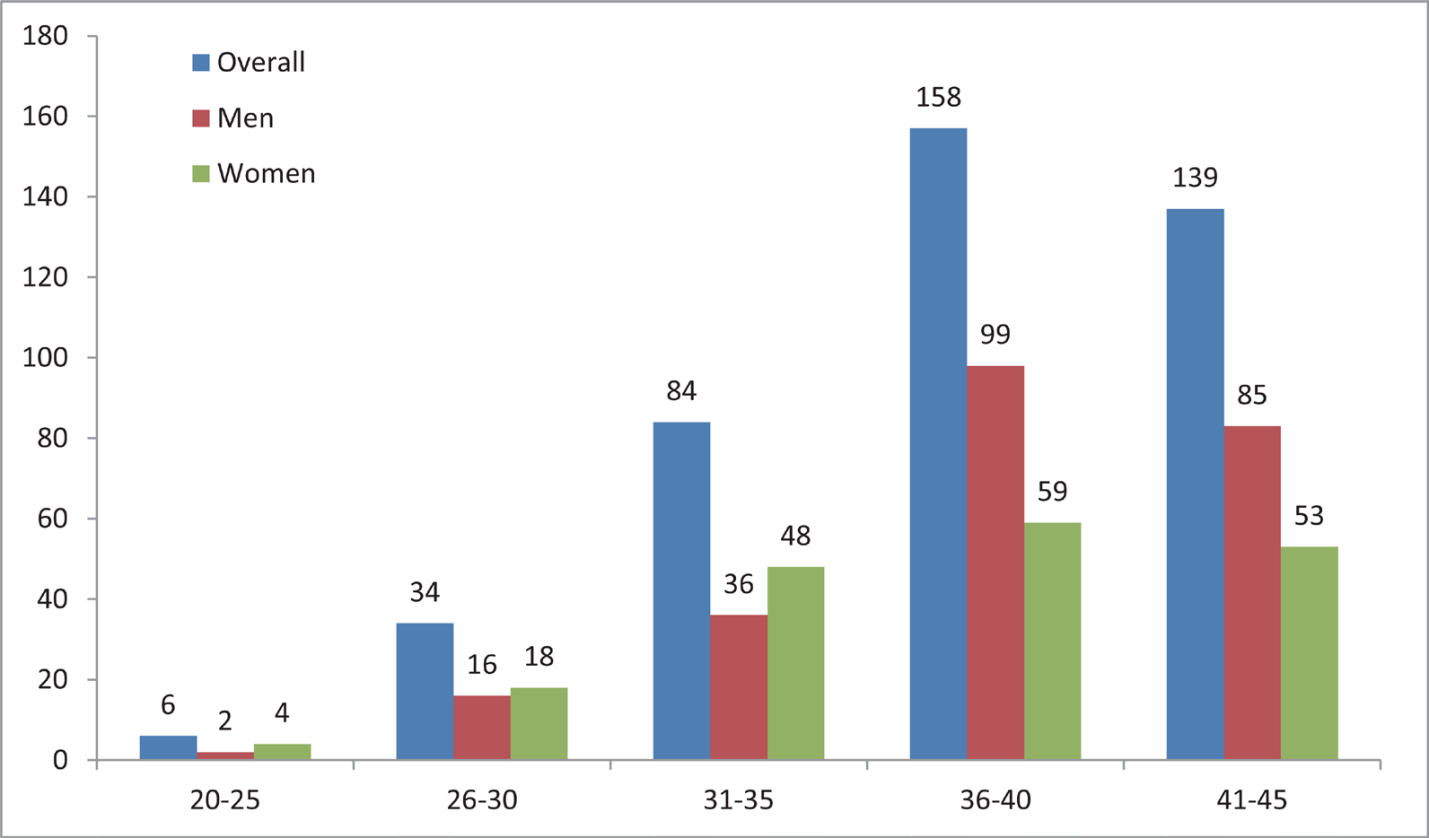
The age and sex distribution of young lung cancer patients

The basic characteristics of the study population were listed in Table 1. Study subjects were aged between 20 and 45 years old, with a median age of 39 years. Patients aged 36 to 40 comprised the largest subgroup (37.6%, Figure 2) while patients aged 20 to 25 years were the smallest subgroup in this cohort (9.5%, Figure 2). Most cases (70%) of young lung cancer were occurred in patients older than 36. Gender distribution showed that lung cancer affected more males than females (56.7% versus 43.3%, Table 1). Current smokers or individuals with a smoking history accounted for more than two thirds (304/420, 72.4%) of the entire group, in which most patients (58.6%) consumed 100–1000 cigarettes per year. Among these smokers, 14.8% had family history of lung cancer.

**Table 1:** Demographic characteristics

	Studied population (*n* = 420, %)
**Median age (year, range)**	39 (20–45)
**Sex**	
Male	238 (56.7)
Female	182 (43.3)
**Occupational exposure^a^**	
General	244 (58.1)
High-risk	176 (41.9)
**Smoking status**	
Non-smoker	116 (27.6)
Formal or current smoker	304 (72.4)
Smoking amount (cigarettes/year)	
≤ 100	39 (9.3)
101–1000	246 (58.6)
> 1000	19 (4.5)
**Family history of lung cancer^b^**	
No	308 (73.3)
Yes	112 (26.7)
**Smoker with family history of lung cancer**	
No	259 (85.2)
Yes	45 (14.8)
**Relatives with tumor^c^**	112 (26.7)
Collateral relative	18 (16.1)
First direct relative	82 (73.2)
Second direct relative	22 (19.6)
**Pathological type**	
Adenocarcinoma	284 (67.6)
Small cell carcinoma	52 (12.4)
Squamous cell carcinoma	31 (7.4)
Mixed adeno-squamous carcinoma	15 (3.6)
Neuroendocrine carcinoma	6 (1.4)
Others	62 (7.6)
**Disease Stage**	
I	47 (11.2)
II	15 (3.6)
III	54 (12.9)
IV	304 (72.4)

^a^High risk population refers to individuals with exposure to soot, dust particles, and toxic gases: cooks, construction workers, traffic policemen, teachers, people exposed to carcinogens, and miners. General population refers to people without high risk exposure.

^b^Refers to a history of lung cancer within 3 generations.

^c^First direct relatives referred to parents, second direct relatives to grandparents, and collateral relatives to uncles and aunts.

Adenocarcinoma was the most common histological type (67.6%) of young lung carcinoma, followed by small cell lung cancer (SCLC) (12.4%). Other histological types such as squamous cell carcinoma (7.4%), adeno-squamous carcinoma (3.6%), neuroendocrine carcinoma (1.4%), and unknown (7.6%) were much less common (Table 1). With respect of staging at diagnosis, most patients had stage IV disease (72.4%), followed by stage III (12.9%), while 14.8% patients had stage I or II disease. A total of 176 (41.9%) subjects were engaged in high-risk occupations that were subjected to soot, dust particles, and toxic gases exposures.

Univariate and multivariate analysis revealed that three factors were associated with the development of lung cancer in young individuals, including the high-risk occupations (OR 5.0, 95% CI 2.0–10; *p* < 0.01), smoking or with a history of smoking (OR 0.4, 95% CI: 0.3–0.8; *p* < 0.01), and family history of lung cancer (OR 1.7, 95% CI 1.1–3.2; *p* < 0.01) (Table 2). In addition, high-risk occupation was the primary factor contributed to lung cancer, with a five-fold risk of developing lung cancer than general occupation.

**Table 2:** Univariate and multivariate analysis for risk factors of incidence in young lung cancer population

Factors	Crude OR (95% CI)	*P*-value	Adjusted OR (95% CI)	*P*-value
**Gender**				
M	1.0	0.18		
F	1.3 (0.7, 1.9)			
**Occupational exposure**				
General	1.0	< 0.01	1.0	< 0.01
High	3.3 (1.7, 10)		5.0 (2.0, 10)	
**Smoking**				
In smoking or had smoking history	1.0	< 0.01	1.0	< 0.01
Non-smoking	0.4 (0.2, 0.6)		0.4 (0.3, 0.8)	
**Family history**				
No	1.0	< 0.01	1.0	< 0.01
Yes	1.6 (0.9, 3.0)		1.7 (1.1, 3.2)	
**Disease type**				
Adenocarcinoma	1.0	0.27		
Small cell carcinoma	0.9 (0.7, 1.3)			
Squamous cell carcinoma	1.5 (0.6, 2.7)			
Others	0.5 (0.2, 1.1)			

**Figure 3:**
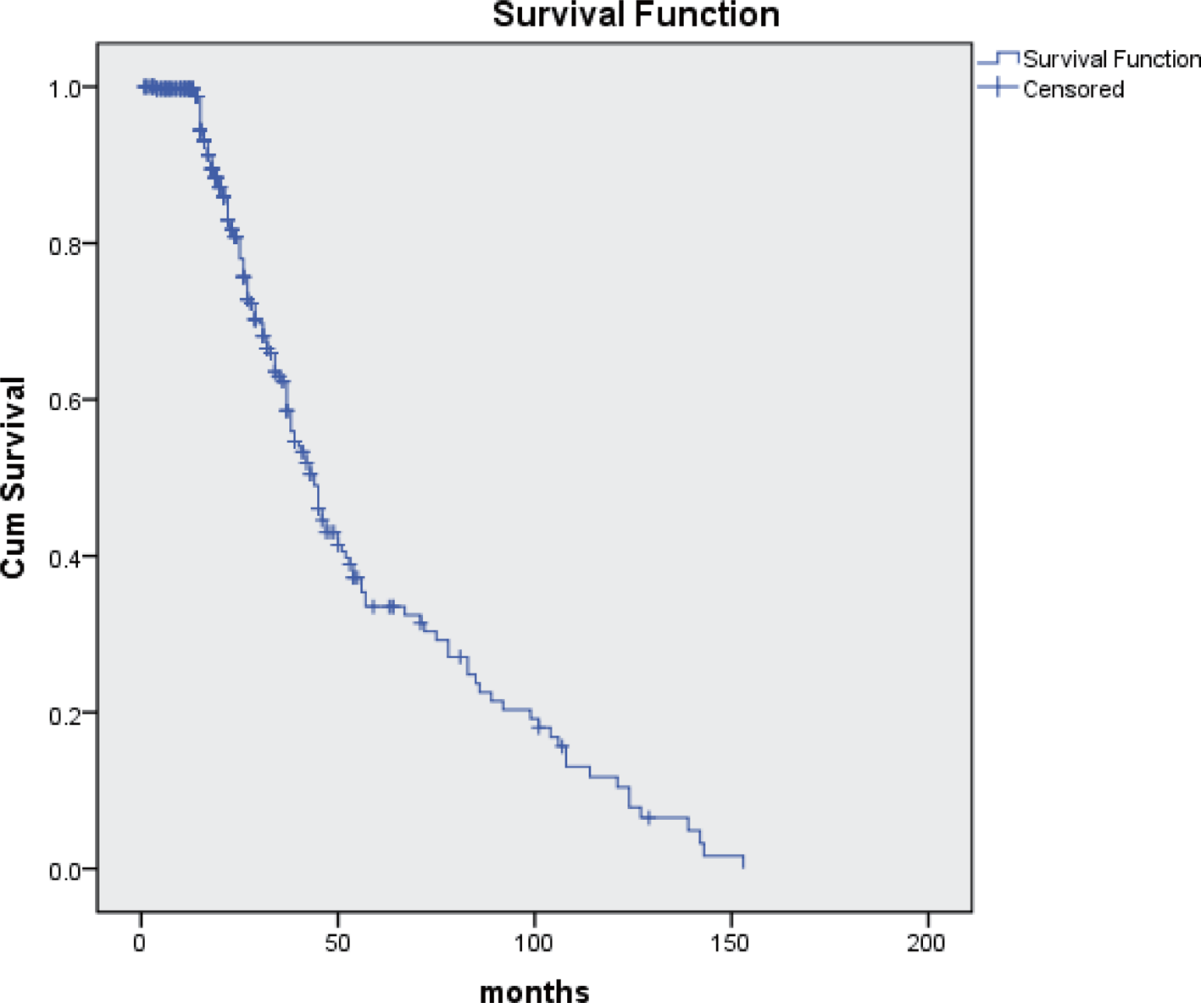
Overall survival in young patients with lung cancer (*n* = 420)

**Figure 4:**
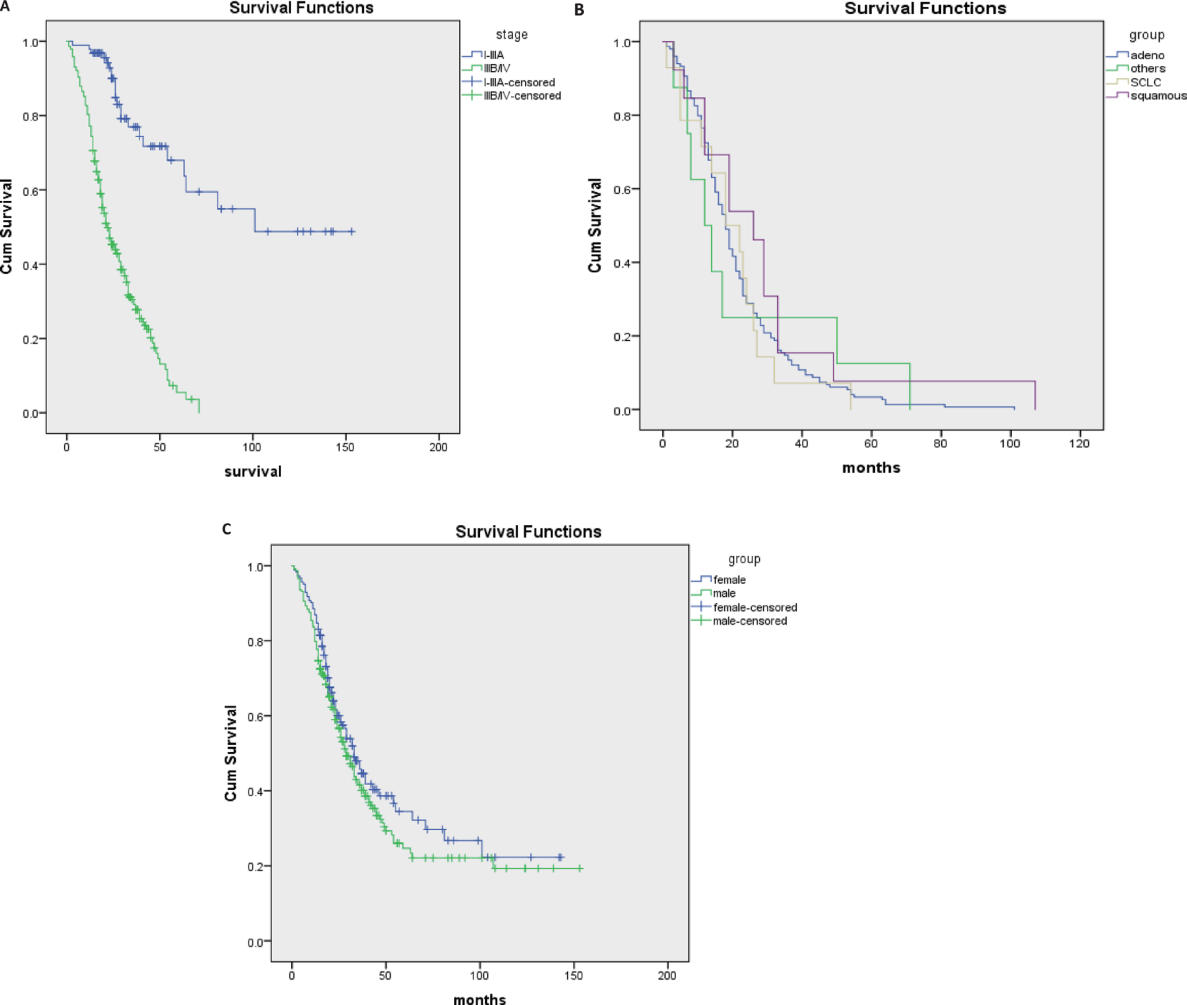
Survival curves for subgroups The plots show the survival curves for patients with different disease stage (**A**) different lung cancer histological type. (**B**) and different gender (**C**).

The median survival of all young patients with lung cancer was 44 months (95% CI 38.9–49; Figure 3). Survival was significantly longer in patients with non-metastatic (stage I–IIIA) disease (101 months; 95% CI 81.7–117.4) when compared to patients with advanced (stage IIIB/IV) disease (22 months; 95% CI 25.0–29.8; *p* < 0.001; Figure 4A). When analyzing patient’s survival stratified by their histological type, we found that patients with adenocarcinoma had the shortest survival as compared with patients with squamous carcinoma (18 months *vs* 26 months; 95% CI 19.2–24.2 months), SCLC or other histological types, however the difference was not statistically significant (Figure 4B) probably due to small number of patients in each group. When analyzing patient’s survival stratified by gender, we noticed that women (33.0 months; 95% CI 46.7–68.3) had longer survival than man (29.0 months; 95% CI 43.4–61.4), but the difference was not statistically significant (Figure 4C).

## DISCUSSION

Our study collected 420 young adults (under age 45) out of 22820 total lung cancer patients, and found a decreasing trend of lung cancer in young population over a 13-year interval (2000–2013). There were several major findings in this study. Firstly, lung cancer frequently occurred in young men than young women, and most patients had a smoking history or family history of lung cancer. Secondly, adenocarcinoma was the most common histological type in young lung cancer patients. Thirdly, the median survival of all young lung cancer patients was 44 months, of which patients with earlier disease stage had longer survival, and the incidence of lung cancer in young population was associated with high exposure risk occupations, family history of lung cancer, and smoking status.

In the current study, a decreasing trend was revealed, which may be attributed to the increasing awareness of risks of smoking in China. The incidence of lung cancer increased beyond age 30 and peaked at age 36–40, which were consistent with previous studies despite various upper limitation of patients age (40, 45 or 50 years) [7, 13]. The gender distribution in our series favored male (56.7% of cases), however, a higher percentage of lung cancer in women was noted in another study [14]. In addition, women develop earlier-age-onset lung cancer compared to men [15, 16]. We also noticed this trend in our study: more women younger than 35 years develop lung cancer when compared to men (38.3% *vs* 23.0%). Most patients (72.4%) were current or former smokers, in which the majority of patients (58.5%) consuming 100–1000 cigarettes per year. We also noted that only 9.3% of cases had a small amount of tobacco smoked (≤ 100 cigarettes per year).

Adenocarcinoma was the most common histological type in our study, presenting 67.6% of all patients. It was also noted as the major type of lung cancers in other aged group. The smoking habits contributed to the high adenocarcinoma incidence in young lung cancer patients [17]. We tentatively believe that person with a family history of smoking or smoking habit will have a higher chance of getting lung related disease and smoking, either in an active or passive way, than person with no family history of smoking or not smoke. On the contrary, person with a family history of lung cancer would have an increased risk of developing lung disease due to genetics but might not significantly affecting life styles such as alcohol drinking and cigarette smoking habits [18, 19]. Our study found that 72.4% patients had stage IV disease at presentation while only 62 (14.8%) stage I and II diseases were recorded. Molecular alterations were seen in lung carcinomas of the young patients when compared with middle-aged and elderly patients [20]. A previous study reported a significantly higher percentage of advanced lung cancer (III/IV stages) in the young population (< 45 years) as compared with that in the elderly group (46% *vs* 16.6%), with stage I lung cancer rarely found in the young population [21]. Yang et al. also demonstrated that stage IIIB/IV lung cancer is more frequently seen in the population less than 50 years old than in the elderly group (72% *vs* 47%) [4]. One possible explanation is that lung cancer in the young population is easy to be misdiagnosed [22], suggesting more attention is needed in the diagnosis for young lung cancer patients.

Smoking status, high-risk occupations/carcinogens, and family history of lung cancer adversely affected the development of lung cancer in the young patients in the present study. Smoking status is directly associated with the development of lung cancer, a five-fold increase in risk was seen in young subjects with a smoking history more than 20 years than non-smoking population [23–25]. Familial aggregation of lung cancer was also existed [26]. Kreuzer et al. indicated a threefold increase in risk of getting lung cancer in younger subjects under 46 years if their relatives had lung cancer [27]. One possible explanation is that younger subjects were more susceptible to develop a smoking habit if family relatives smoke. We also found that 14.8% of former or current smoker in this study had family history of lung cancer. The certainty of enhanced risk for genetic susceptibility in young patient population should be explored in our future study. In addition, risk factors differed between women and men. Higher exposure to other risk factors, such as work-related air pollution or carcinogens, may result in an increased susceptibility in men [27, 28], while passive smoking and household exposure was demonstrated as important causes of the development of lung cancer in women according to recent studies [29–31].

Survival for lung cancer was strongly dependent on diagnosed age (young patients were always related with advanced disease and their survival was greater than older patients). A European study demonstrated that estimated 5-year relative survival was 18% in patients aged 15–44 years while only 6% in patients older than 75 [32]. The SEER study also reported a significantly better 5-year survival rate in the younger group compared with that in the older group (16.1% versus 13.4%, *P* < 0.001) [13]. However, the recruiting period of these studies were 1990–1994 and 1973–1992, respectively, while great improvements of diagnosis and treatments took place in the recent decades. In the current study, the median survival of all young patients with lung cancer was 44 months. Better survival (101 months) was seen in patients with early disease stage (I–IIIA), while only 22 months was seen in stage IIIB/IV patients. Patients with squamous cell carcinoma had a better survival although no significance of survival was found among different histological types. Several factors should be considered when interpreting these results: only 420 were enrolled in this study, the relative small sample size would induce selection bias; 14.8% of cases had stage I or II disease, which are associated with better survival [33]; in addition, young patients are more likely to receive aggressive multimodality therapy. Our study analyzed data from young population diagnosed between 2000 and 2013. During this time, some advancements of diagnosis and treatment were introduced into clinical practice. Our study reflects the clinical outcome after these advancements introduced into clinical practice.

Our study has several limitations: the results were limited by the lack of control group; the small sample size and the study population restricted to two centers would induce selection bias and referral bias to some extent. Despite increasing recognition on the importance of biomarkers, we failed to collect the biological data since biomarker analysis has not universalized in clinical practice until very recent years and our patients were from areas nationwide in which some had few access to biomarker testing, further studies are warranted for targetable driver mutations in young patients. A very recent retrospective study published on JAMA Oncology has reported that younger patients (less than 40 years old) have high frequency of targetable mutations (EGFR mutations, ALK and ROS1 translocations) [34]. However, only 81 patients in that study were less than 40 years old. Therefore, studies with larger young lung cancer patient number are needed to make the conclusions more valid, and we would like to include large scale genetic analysis of young lung cancer patients in China into our future work. Furthermore, our study did not include the interactions of all possible risk factors on young lung cancer patients, this was due to the immature electronic hospital information system at this study starts, and most of data were collected by paper-based system. Despite of these limitations, this study still holds some source value since little data is available in Chinese young lung cancer patients.

In summary, our study demonstrated a decline trend of lung cancer in young patients. The overall prognosis for lung cancer patients remains poor, with smoking, family history of lung cancer, and high-risk occupations/carcinogens were the main adverse factors. Efforts should be made on the tobacco products elimination, and the occupational protection. Family history of lung cancer was also identified as a risk factor; therefore, further studies are warranted to detect the possible underlying biological mechanism. In addition, young population deserves more studies, especially studies on searching targetable driver mutations.

## MATERIALS AND METHODS

### Study design and patients

Patients aged 18 to 45 years who had pathologically confirmed lung cancer were identified from prospectively maintained database in Department of Thoracic Surgery of People’s Hospital of Peking University, and Departments of Pulmonary Tumor and Respiration in the 307 Hospital Affiliated to the Academy of Military Medical Sciences between 2000 and 2013. The study protocols were approved by the Institutional Review Board of both Hospitals. Written consent was waived because only non-identifiable information was used. The majority of patients enrolled in this study were ethnically Han Chinese. Because of the minute number of patients with other ethnicities, the impacts of different susceptible genetic background could be ignored.

### Data collection

Parameters including epidemiologic data, gender, age, smoking status, smoking history, histological classification, disease stage, occupations, family history of cancer, and survival information were collected from database.

Smoking status was divided into current or former smoker versus never-smoker. Never-smokers were defined as individuals who report smoking less than 100 cigarettes per lifetime. Former smokers were defined as smoking cessation at least 2 years prior to diagnosis of lung cancer. Patients diagnosed before 2009 were re-staged according to the TNM classification by American Joint Committee on Cancer. High risk occupational exposure population were defined as individuals with exposure to soot, dust particles, toxic gases such as cooks, carcinogens, and miners. No family history of cancer referred to individuals who have no history of cancer within the family for three generations. First-degree relatives referred to parents, second-degree relatives to grandparents, and collateral relatives to uncles and aunts. Overall survival was defined as the time from the date of diagnosis to the date of death.

### Statistical analysis

A multivariable logistic regression analysis model was employed to identify risk factors associated with lung cancer with a stepwise approach for variable selection including gender, histological type, and disease stages (stages I–IIIA and stages IIIB–IV). Variables exhibiting a statistically significant association (*p* < 0.05) with lung cancer in univariate analyses were further evaluated by multivariate analyses. Kaplan-Meier method was used to estimate the survival in all young lung cancer patients and in subgroups. Two-sided *p* < 0.05 was considered statistically significant. Analyses were carried out using SPSS version 23.0 (SPSS, Chicago, IL, USA).
